# You Eat with Your Eyes: Framing of Food Choice Options Affects Decision Conflict and Visual Attention in Food Choice Task

**DOI:** 10.3390/nu16193343

**Published:** 2024-10-01

**Authors:** Ulrike Senftleben, Johanna Kruse, Stefan Scherbaum, Franziska M. Korb

**Affiliations:** Faculty of Psychology, TUD Dresden University of Technology, 01062 Dresden, Germany; johanna.kruse@tu-dresden.de (J.K.); stefan.scherbaum@tu-dresden.de (S.S.); franziska.korb@tu-dresden.de (F.M.K.)

**Keywords:** food choice, decision-making, decision conflict, eye-tracking, attentional processes

## Abstract

**Background/Objectives:** Frequent poor dietary choices can have significant consequences. To understand the underlying decision-making processes, most food choice tasks present a binary choice between a tasty but less healthy option and a healthy but less tasty option. It is assumed that people come to a decision by trading off the respective health and taste values. However, it is unclear whether and to what extent food choice goes beyond this. **Methods:** We use a novel eye-tracking experiment where we compare a typical food choice task (image condition) with an abstract value-based decision-making task using pre-matched percentages of health and taste (text condition; e.g., 10% healthy and 80% tasty) in 78 participants. **Results:** We find a higher frequency of unhealthy choices and reduced response times in the image condition compared to the text condition, suggesting more impulsive decision making. The eye-tracking analysis shows that, in the text condition, the item corresponding to the subsequent choice receives more attention than the alternative option, whereas in the image condition this only applies to the healthy item. **Conclusions:** Our findings suggest that decision-making in typical food choice tasks goes beyond a mere value-based trade-off. These differences could be due to the involvement of different attentional processes in typical food choice tasks or due to the modality of stimulus presentation. These results could help to understand why people prefer tasty but unhealthy food options even when health is important to them.

## 1. Introduction

In everyday life, we frequently have to choose between options offering immediate rewards and those providing benefits in the future. Consider, for instance, the decision-making process at a cafeteria when selecting lunch, where one must decide between a burger and a salad. While the burger may offer immediate satisfaction of taste cravings, the salad represents a healthier and more advantageous choice in the long term. Such decisions, characterized by subjective values and potential costs for each option without an objectively correct answer, are termed value-based decisions [1]. Each option’s different benefits and costs create a conflict during the decision-making process [2,3,4]. Although there is no objectively right or wrong decision in this type of conflict, consistently favoring immediate rewards over long-term benefits can lead to significant harmful consequences. This is a particular challenge for dietary decisions, where regularly preferring immediately rewarding but unhealthy foods can contribute to obesity, which in turn is associated with adverse outcomes such as depression, anxiety, hypertension, type 2 diabetes mellitus, and substantial healthcare expenditures [5,6]. Despite individuals recognizing that a healthier diet is preferable, many attempts to adhere to a diet remain unsuccessful [7]. Therefore, it is crucial to comprehend how the decision conflict between healthy but less tasty and tasty but unhealthy food options is processed and how it can be influenced in favor of options with long-term benefits.

In experimental psychology, this conflict is typically investigated using a binary food choice task. In this task, participants must decide between one food option that is tasty but less healthy (e.g., a burger) and another food option that is healthy but less tasty (e.g., a salad). The food options are typically presented as images [8,9,10,11]. In order to come to a decision, it is assumed that participants trade-off the taste and health properties of both food items. Since taste is processed faster [11] and is more immediately rewarding, choosing the healthy option is considered to require self-control [12]. The amount of healthier choices can be increased by manipulations such as providing additional information in the form of caloric information [9] or by presenting explicit or implicit cues that focus on long-term goals and consequences [13,14,15] or emphasize the tastiness of healthy options [16]. When considering the trade-off between taste and health that occurs during the decision-making process, these manipulations could affect decision-making by increasing the relative weights of health and taste attributes and therefore introducing a stronger focus on the healthy option [17]. Similar effects have been found for other types of value-based decisions, such as intertemporal choice, where participants have to choose between an immediate but smaller monetary reward and a delayed but large monetary reward e.g., [10]. However, there is an important difference to consider when comparing food choice tasks to other types of value-based decision-making. In food choice tasks, the health and taste attributes are not presented explicitly; rather, food options are presented as an image and participants assign their own subjective health and taste values to each option. Therefore, it is unclear how this trade-off between health and taste occurs and how other factors determine the decision-making process in food choice.

Hence, exploring potential differences between a value-based trade-off and typical food choice tasks can provide new insights. This can be achieved by comparing a typical food choice task (e.g., choose between an apple and a chocolate bar) and an explicit value-based task, where attribute values of taste and health are presented separately for each food item (i.e., choose between an option that is 80% healthy and 40% tasty and an option that is 40% healthy and 80% tasty). In such a comparison, differences may arise even just through the different presentation modalities (image vs. text). This is because humans can semantically process information faster if it is presented pictorially instead of in words [18,19], which can result in more impulsive decisions [20]. However, regardless of how the decision is framed (as images or as text), participants have to weigh which option they find more valuable overall (i.e., they have to trade-off health and taste) to come to a decision. This should induce decision conflict, particularly when the difference in subjective value between both options is small [8,21]. Decisions with a small difference in value between the options, i.e., where both options are equally liked, are considered more difficult and are associated with longer response times than decisions with a high difference in value, i.e., where there is a strong preference for one option. In a previous study, we showed that decision conflict and difficulty effects occur in both food choices (presented as images) and in abstract value-based trade-offs of health and taste (presented as text), but that cognitive processing might differ [22].

In addition, further insights into the decision-making process can be gained by tracking the underlying attentional processes. This can be achieved by recording eye movements, which serve as indicator of overt visual attention. As stated by the gaze-bias theory [23], people spend more time looking at the decision option that they eventually end up choosing. Considering food choices, it has been shown that the chosen option has more fixations and longer dwell times compared to the alternative option [17,24,25,26], i.e., that more attention is paid to it. The visual attention an object attracts is influenced by top-down and bottom-up processes [27]. Top-down (goal-oriented) processes rely on the voluntary allocation of attention to objects relevant to the goal or task currently being pursued [28]. In contrast, bottom-up (stimulus-oriented) processes are based on visual features (e.g., color, size, texture) or internal attributes of stimuli that automatically attract someone’s attention [29]. The attention an object captures can be changed by influencing these processes. Possible ways to influence top-down attention are, for example, health goals and health logos on food. Both reinforce the importance of a healthy diet. Previous research showed that health goal primes increase the probability of choosing a healthy food item and the dwell times on low-energy food products [30]. Bottom-up attention can be influenced by modifying the stimulus properties. For example, visual salience can affect decision making through its effects on attention by biasing choices towards more visually salient options [31,32]. These results also apply to more realistic situations like the supermarket [33]. As stated above, the modality of the stimulus is also an important factor influencing the decision-making process, as it affects bottom-up attention. Freijy, Mullan, and Sharpe [34] used a dot-probe task to calculate an attentional bias toward high-caloric and low-caloric food items presented as pictures or words. The dot-probe task involves short presentations of pairs of images or words on the screen. Subsequently, a probe (typically represented by a dot or asterisk) emerges at the location of one of the previously displayed stimuli. Participants are asked to indicate the probe’s location as fast as possible. This procedure allows the discrimination between attention directed toward stimuli and attention oriented away from stimuli, offering a more refined assessment of attentional allocation. Consequently, an attentional bias toward target stimuli is evidenced by the quicker detection of probes replacing such stimuli. In contrast, delayed detection of probes replacing such stimuli indicates attentional avoidance of target stimuli. Freijy et al. (2014) found an attentional bias toward high-caloric foods when the stimuli were presented as pictures and toward low-caloric foods when stimuli were presented as words. This finding suggests that the modality of the stimulus can influence attentional biases. Although taste and health attributes were not explicitly evaluated, high-caloric foods are often associated with taste, whereas low-caloric foods are connected to health [35,36]. However, it is still unknown whether and how attentional biases affect the actual decision-making in process in food choices.

Therefore, the aim of our study is to close this gap and to understand if and how attentional processing in a typical food choice task deviates from a value-based trade-off. This could have powerful implications for understanding why people behave inconsistently with their own dietary beliefs and goals, for example preferring tasty but unhealthy food even when health is an important attribute for them. Identifying potential attentional biases can hence help to influence the decision conflict and promote healthier food choices and, thus, a healthy lifestyle. In the present study, we combined eye-tracking with a novel experimental setup that we introduced in our previous study [22]. In the task, participants completed both a typical food choice consisting of food images (subsequently referred to as image condition) and an abstract value-based decision task consisting of options presented as a percentage of perceived taste and health values (subsequently referred to as text condition). We expected to replicate the following behavioral effects from our previous study [22]:

**H1:**  *The proportion of healthy choices is lower in the image condition than the text condition.*

**H2:**  *Response times in the image condition are faster than in the text condition.*

**H3a:**  *For both conditions, response times in healthy choices are longer than in unhealthy choices.*

**H3b:**  *The effect of choice type on response times differs both conditions.*

**H4a:**  *For both conditions, response times for difficult decisions (i.e., decisions with a small value difference between the two decision options) are longer than for easy decisions.*

**H4b:**  *The effect of difficulty on response times differs between both conditions.*

In addition, we derived the following eye-tracking hypotheses from the literature described above:

**H5a:**  *For both conditions, there are longer dwell times and more fixations on the decision option that participants eventually choose (i.e., the healthy item in healthy choices and the tasty option in tasty choices) (as predicted by the gaze-bias theory* [23] *and previous findings in food choice tasks* [17,24,25,26]*)*.

**H5b:**  *These effects of dwell time and fixations differ between both conditions (as derived from attentional differences for high- vs low-caloric food* [34] *and from effects of visual salience* [31,32]*)*.

## 2. Materials and Methods

### 2.1. Data Statement

We report how we determined our sample size, all data exclusions (if any), all manipulations, and all measures in the study. All data and analysis scripts are openly accessible at https://osf.io/7qmdc/. The study was not preregistered.

### 2.2. Participants

We based our sample size estimation on a small effect size of ηp^2^ = 0.01. Using MorePower 6.0.4 [37], the power analysis yielded a sample size of 74 participants for a 2 × 2 × 2 repeated measures ANOVA with a power of 0.8. To compensate for possible drop-out, we aimed for a final sample size of 81 participants (74 participants + 10%). We recruited participants from the ORSEE-based database [38] of the Faculty of Psychology of the Dresden University of Technology, Dresden, Germany. Participants had to meet the following criteria: 18–35 years of age, no weight-loss-oriented restricting diet, no dyschromatopsia, normal or corrected-to-normal vision, no other eye diseases (e.g., strabismus), currently not in psychotherapeutic treatment, and German language skills at native level. A total of 91 participants took part in the experiment (64 female, 24 male, 3 not specified, mean age = 23.69 years, *SD* = 4.16 years). Due to technical difficulties, eight participants could not complete the experiment, and two additional participants had to be excluded during data processing (see data preprocessing). The final sample consisted of 81 participants (59 female, 20 male, 2 not specified, mean age = 23.69 years, *SD* = 4.29 years). For three participants, the behavioral data were recorded but no eye-tracking data were recorded. Therefore, the final sample for analyzing the eye-tracking data included 78 participants (56 female, 20 male, 2 not specified, mean age = 23.69 years, *SD* = 4.24 years). All participants were remunerated with either a payment of EUR 12 (8 EUR/h), or received course credit in exchange for their involvement in the study. Prior to engaging in the experiment, all participants provided informed consent. The study was performed in accordance with the Declaration of Helsinki and was approved by the ethics committee of Dresden University of Technology, Germany (IRB00001473/EK47022016).

### 2.3. Apparatus and Stimuli

We used an EyeLink 1000 desk-mounted eye tracker (SR Research, Kanata, ON, Canada) to record eye movements, with a reported average accuracy between 0.25° and 0.50° of visual angle and a root mean square resolution of 0.01° (www.sr-research.com). We tracked the movements of the participant’s right eye using the combined pupil and corneal reflection setting at 500 Hz sampling rate. A chin rest was employed to ensure minimal interference from head movements and to maintain a consistent eye-to-monitor distance of 60 cm. Before each block of the experimental task, we ran a nine-point grid calibration using a grid of three horizontal positions and three vertical positions and a drift correction. The eye-tracker was re-calibrated if validation yielded an average error of over 1°. Overall, the eye-tracker achieved an average error across participants of 0.38° (*SD* = 0.11°) with a maximum error of 0.85° on average (*SD* = 0.37°).

**Figure 1:**
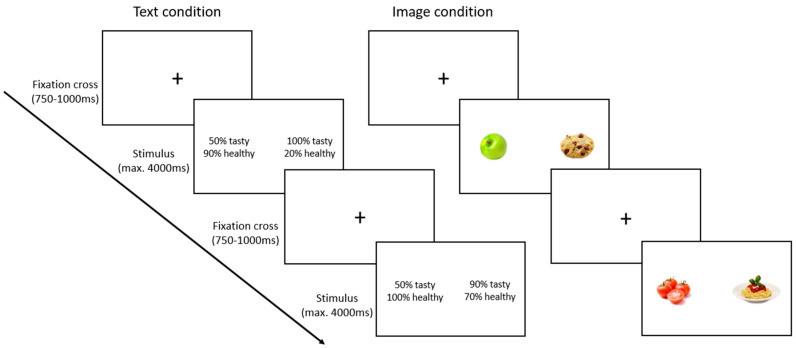
Schematic of the procedure during the text condition (**left**) and image condition (**right**). Each trial began with a fixation cross. Afterwards, stimuli were presented and participants had maximal 4000 ms to choose which option they preferred.

The task was presented on a 24-inch screen (1920 × 1080 pixels, 72 Hz). We used the Psychophysics Toolbox 3 [39] in Matlab 2018b (the Mathworks Inc., Natick, MA, USA) on a Windows 10 computer to present the experiment. Participants used a wired USB keyboard to make their responses (QWERTZ-layout, ‘Y’ for left and ‘M’ for right option). In the image condition, we employed a set of colored food images (300 × 225 pixels) sourced from the Food-pics image database [40]. In the text condition, we used numbers as percentages and the German words ‘gesund’ (meaning healthy) or ‘lecker’ (meaning tasty) to display taste and health values for each option. The text was presented using the font type ‘Calibri’ in font size 36. For each option, both attributes (taste and health percentages) were displayed together, with a combined size of 176 × 80 pixels (see Figure 1).

### 2.4. Procedure

First, participants provided informed consent and completed a brief pre-experiment questionnaire, disclosing information about their age, gender, current health status, and mood. Additionally, they underwent a vision assessment to confirm normal or corrected-to-normal vision and assess color perception. Subsequently, each participant was presented with two sets of materials printed on paper. The first set featured 274 food images [40], encompassing various items ranging from vegetables and fruits to indulgent treats like chocolate and candy and fast food items like hamburgers or pizza. The second set comprised 28 abstract textual descriptions detailing combinations of healthiness and tastiness values represented as percentages (e.g., 20% healthy and 80% tasty). Participants were asked to associate specific food items from the first set with the abstract value combinations presented in the second set. For instance, each participant selected a food item they considered 90% healthy and 10% tasty. These personally chosen food images then formed the basis for the individualized stimulus sets used for the trials in the main experiment. Next, participants were informed about the eye-tracking setup. If necessary, the chair height was changed so the participants could comfortably position their heads on the chin rest.

Subsequently, participants engaged in a food choice task requiring them to indicate their preference between two presented food items. The decision task comprised four blocks, each containing 148 trials (for task details, refer to the task design section), with self-paced breaks provided between these blocks. Each trial began with the display of a fixation cross, followed by the presentation of the stimuli (see Figure 1). Following the onset of the stimulus, participants had to make their decision within four seconds. Upon responding or upon the expiration of this time limit, the subsequent trial commenced, marked by the appearance of another fixation cross. The intertrial interval (ITI) was randomized between 750 ms and 1000 ms. To familiarize themselves with the task, participants completed a tutorial of 30 practice trials before performing the actual decision task. The eye-tracking system was validated (and re-calibrated if necessary) after the tutorial and between blocks.

After the food choice task, participants completed a brief post-experiment questionnaire, which inquired about their current emotional state, mood, and conjecture regarding the purpose of the experiment. Overall, the experiment had a duration of approximately 1.5 h.

### 2.5. Task Design

We implemented a within-subjects factorial design with the factors *presentation format* and *trial type*. The *presentation format* consisted of two levels: percentage-based descriptions (referred to as the text condition) or images of food items (the image condition), which were manipulated on a block-wise level. In the text condition, food items were described using numerical percentages for their health and taste attributes. For instance, participants were presented with a choice between an item that was 90% healthy and 50% tasty and another item that was 20% healthy and 100% tasty. In the image condition, food items were represented by images (e.g., an apple and a cookie) (see Figure 1).

The *trial type* consisted of conflict trials and control trials. Conflict trials required self-control by offering the choice between one food option with a higher health percentage (termed the “healthy item”) and another food option with a higher taste percentage (termed the “tasty item”). The health value of the healthy item was either 90% or 100%, and its taste value was either 10%, 20%, 40%, 50%, 70%, or 80%, yielding twelve unique combinations. The taste value of the tasty item was either 90% or 100% and its health value was 10%, 20%, 40%, 50%, 70%, or 80%, resulting in twelve additional combinations. This procedure yielded a total of 144 unique conflict trials. Control trials were designed to assess the consistency of participants’ decision making. They consisted of one food item that was both healthy and tasty and one item that was neither healthy nor tasty. The healthy and tasty items were either 90% healthy and 100% tasty or 100% healthy and 90% tasty. The items that were neither healthy nor tasty were 10% healthy and 20% tasty or 20% healthy and 10% tasty. This yielded a total of four control trials. All trials (conflict and control) were presented in four blocks, with each block containing all trials in randomized order (4 × 148 = 592 trials). Two of these blocks presented images as stimuli and two blocks presented percentages (order balanced across participants). Additionally, we counterbalanced the positions of the healthy food item (right or left side of the screen) and, in the percentage condition, the position of the attributes (health value above taste value and vice versa) across participants.

To assess decision difficulty, we calculated the difference between the sum of health and taste values for each food item. These differences could be 0, 10, 20, 30, 40, 50, 60, 70, or 80. Decisions with differences falling within the range of 0 to 20 were categorized as difficult, while those within the range of 60 to 80 were considered easy.

### 2.6. Data Preprocessing

As a consistency check as to whether participants made valid choices, we analyzed choices in control trials (where we expected participants to choose the item that was both tasty and healthy instead of the item that was neither). Two participants were excluded due to their number of incorrect choices in control trials (25% vs. 50%). Moreover, we calculated the mean overall response times for each participant. One participant had very low response times (*M* = 0.64 s; *SD* = 0.18 s; *Min* = 0.26 s; *Max* = 1.53 s). However, since the exclusion of this participant did not influence the quality of the results, we decided to retain this dataset in the analysis.

### 2.7. Data Analysis of Behavioral Data

We used MATLAB 2020b for data processing and analysis. For statistical analyses, we utilized JASP version 0.10.2.0 [41]. We analyzed participants’ healthy choice percentage in conflict trials using a repeated measures analysis of variance (rmANOVA) with the factors *Visualization* (text; image) and *Difficulty* (easy; difficult). We analyzed response times in conflict trials using an rmANOVA with the factors *Visualization* (text; image), *Difficulty* (easy; difficult), and *Choice* (healthy; unhealthy). For the analysis of the eye-tracking data, we also calculated an rmANOVA including the within factors *Visualization* (text; image), *Choice* (healthy; unhealthy), and *Item* (healthy; tasty). Furthermore, as an exploratory analysis, we calculated an rmANOVA including the within factors *Visualization* (text; image), *Difficulty* (easy; difficult), and *Item* (healthy; tasty).

## 3. Results

### 3.1. Behavioral Results

**Figure 2:**
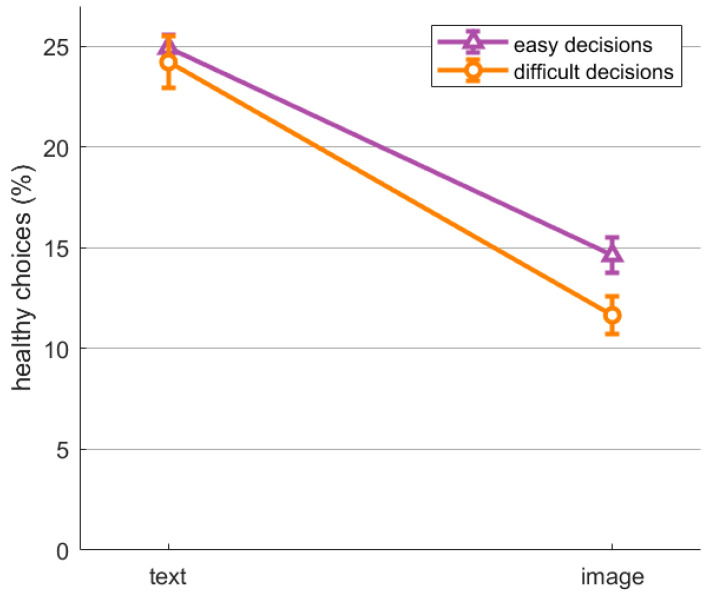
Percentage of healthy choices for each condition (text vs. image) and difficulty level (easy vs. difficult). Error bars represent standard errors.

First, we examined participants’ choice behavior. We expected a reduced proportion of healthy choices in the image condition compared to the text condition (H1). Additionally, we aimed to investigate the effects of difficulty as an exploratory analysis. To analyze the impact of both these factors, we employed a 2 × 2 rmANOVA with the within factors *Visualization* (image; text) and *Difficulty* (easy; difficult) on the proportion of healthy choices. We found a significant main effect *Visualization*, *F*(1,80) = 105.55, *p* < 0.001, η^2^ = 0.31. As predicted, participants exhibited a decreased proportion of healthy choices in the image condition (*M* = 13.25%, *SD* = 8.11%) in comparison to the text condition (*M* = 24.60%, *SD* = 9.03%), as visually represented in Figure 2. Furthermore, we found a significant main effect for *Difficulty*, *F*(1,80) = 11.56, *p* = 0.001, η^2^ = 0.01. Participants chose the healthy option more often in easy decisions (*M* = 19.91%, *SD* = 8.53%) than in difficult decisions (*M* = 17.94%, *SD* = 11.72%). The interaction effect Visualization*Difficulty was not significant but showed a trend, *F*(1,80) = 3.67, *p* = 0.06, η^2^ = 0.003.

Subsequently, we analyzed participants’ response times. We expected lower response times in the image condition compared to the text condition (H2). Additionally, we expected that responses would be faster for selecting unhealthy options as opposed to healthy ones (H3a) and for making easy decisions in contrast to difficult ones (H4a). We further expected interactions between the conditions and choice type (H3b) as well as condition and difficulty (H4b). We conducted an rmANOVA with the within factors *Visualization* (image; text), *Choice* (healthy; unhealthy), and *Difficulty* (easy; difficult). All three main effects reached significance (*Visualization*, *F*(1,74) = 244.44, *p* < 0.001, η^2^ = 0.40; *Difficulty*, *F*(1,74) = 142.79, *p* < 0.001, η^2^ = 0.02; *Choice*, *F*(1,74) = 21.59, *p* < 0.001, η^2^ = 0.01). 

**Figure 3:**
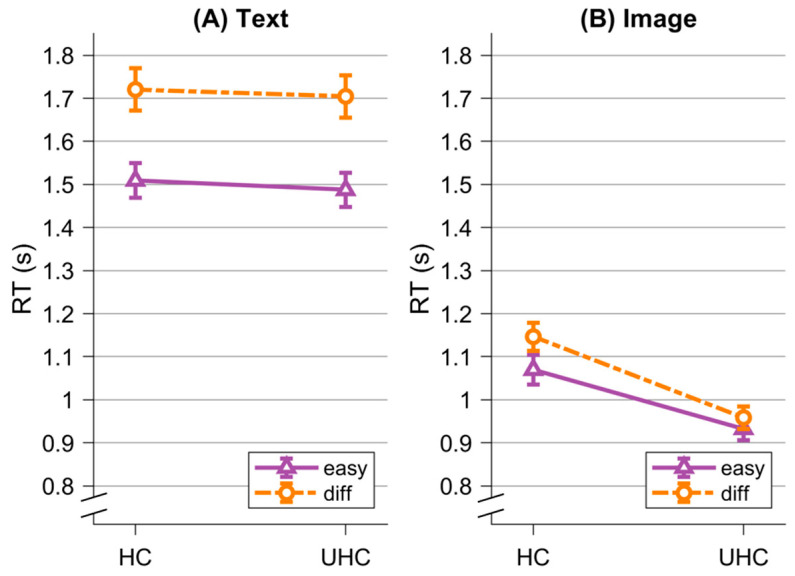
Response times (RT) for easy decisions (easy) and difficult decisions (diff), divided into healthy choices (HC) and unhealthy choices (UHC). (**A**) shows results for the text condition, while (**B**) shows results for the image condition. Error bars represent standard errors.

As expected, response times were shorter for trials presented in image format (*M* = 0.98 s, *SD* = 0.22 s) in comparison to text-based trials (*M* = 1.62 s, *SD* = 0.40 s), supporting H2. Similarly, participants exhibited faster responses for unhealthy choices (*M* = 1.25 s, *SD* = 0.29 s) when compared to healthy choices (*M* = 1.47 s, *SD* = 0.34 s), supporting H3a, as well as for easy decisions (*M* = 1.24 s, *SD* = 0.27 s) in comparison to difficult decisions (*M* = 1.35 s, *SD* = 0.31 s), supporting H3b (see Figure 3). Moreover, we observed a significant interaction effect for Visualization * Difficulty, *F*(1,74) = 72.59, *p* < 0.001, η^2^ = 0.01 and Visualization * Choice, *F*(1,74) = 21.25, *p* < 0.001, η^2^ = 0.006, supporting H3b and H4, respectively. Descriptively, the disparity in response times between difficult and easy decisions was more pronounced in the text condition than in the image condition, where the difference was nearly negligible. There was also a difference between healthy and unhealthy choices in the image condition, with longer response times for healthy compared to unhealthy choices. This difference was negligible in the text condition. The other two interaction effects were not significant (Difficulty * Choice, *F*(1,74) = 3.03, *p* = 0.086, η^2^ = 0.00; Visualization * Difficulty * Choice, *F*(1,74) = 3.44, *p* = 0.07, η^2^ = 0.00). Mean response times and their standard deviations are detailed in Table 1.

### 3.2. Eye-Tracking Results

**Figure 4:**
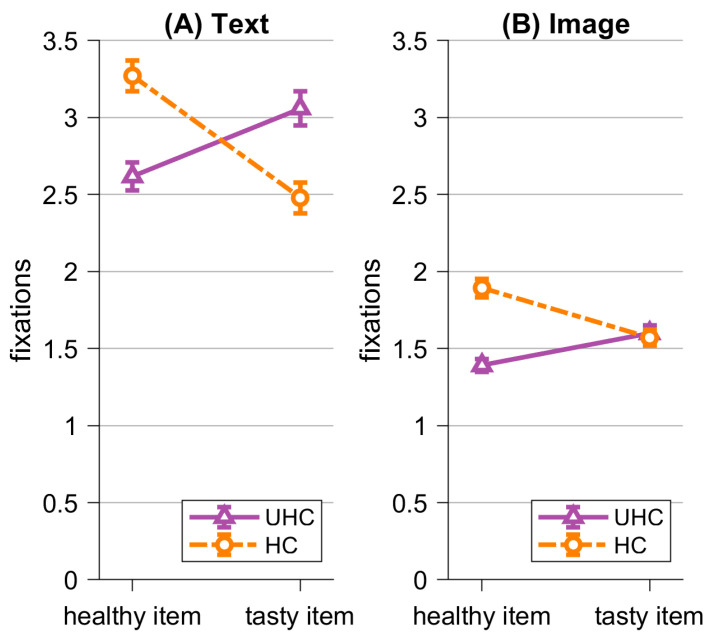
Mean fixations on healthy Item (healthy) and tasty Item (tasty), divided into healthy choices (HC) and unhealthy choices (UHC). (**A**) shows results for the text condition, while (**B**) shows results for the image condition. Error bars represent standard errors.

Next, we analyzed participants’ eye-tracking data. We expected more fixations of the healthy item compared to the tasty item for healthy choice, and we expected more fixations on the tasty item compared to the healthy item for unhealthy choices (H5a). We also expected an interaction with condition (H5b). We calculated an rmANOVA with the within factors *Visualization* (image; text), *Item* (healthy; tasty), and *Choice* (healthy; unhealthy). We found a significant main effect for *Visualization*, *F*(1,77) = 241.35, *p* < 0.001, η^2^ = 0.41. Participants looked more often at the items in the text condition (*M* = 2.77 fixations, *SD* = 0.88 fixations) as compared to the image condition (*M* = 1.52 fixations, *SD* = 0.41 fixations), as displayed in Figure 4. Furthermore, we found a significant interaction effect Visualization * Item * Choice, *F*(1,77) = 4.83, *p* < 0.001, η^2^ = 0.008, supporting H5b. Descriptively, in the text condition there were more fixations on the item that was eventually chosen. That is, the healthy item was fixated more often in healthy choices compared to unhealthy choices, and the tasty item was fixated more often in unhealthy choices compared to healthy choices. This is in line with our hypothesis (H5a). In the image condition, this difference only existed for the healthy item and not for the tasty item. This pattern contradicts our expectations (H5a). All other effects were significant (*Choice, F*(1,77) = 2.93, *p* = < 0.001, η^2^ = 0.005; *Item, F*(1,77) = 5.30, *p* = 0.024, η^2^ = 0.004; Item * Choice, *F*(1,77) = 310.65, *p* < 0.001, η^2^ = 0.05; Visualization * Choice, *F*(1,77) = 23.69, *p* < 0.001, η^2^ = 0.003), except for Visualization * Item, *F*(1,77) = 0.055, *p* = 0.10, η^2^ = 0.00. Mean fixations and their standard deviations are detailed in Table 2. We ran the same analysis for dwell times and found the same qualitative pattern of results (for details, see Supplement S1).

**Figure 5:**
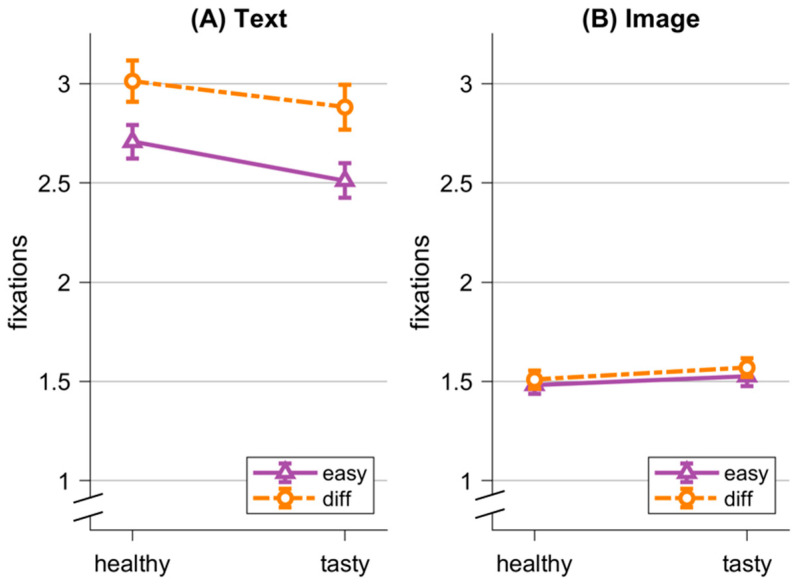
Fixations on healthy Item (healthy) and tasty Item (tasty), divided into easy decisions (easy) and difficult decisions (diff). (**A**) shows results for the text condition, while (**B**) shows results for the image condition. Error bars represent standard errors.

As an exploratory analysis, we examined effects of difficulty on visual attention (see Figure 5). We conducted an rmANOVA with the within factors *Visualization* (image; text), *Item* (healthy; tasty), and *Difficulty* (easy; difficult). We found a significant main effect for *Visualization*, *F*(1,77) = 300.23, *p* < 0.001, η^2^ = 0.46. Participants looked more often at the items in the text condition (M = 2.77 fixations, *SD* = 0.88 fixations) as compared to the image condition (M = 1.52 fixations, *SD* = 0.41 fixations). Furthermore, we found a significant interaction effect for Visualization * Difficulty, *F*(1,77) = 77.65, *p* < 0.001, η^2^ = 0.007. Descriptively, participants looked more often at the difficult item compared to the easy item in the text condition, whereas there was no difference in the image condition. The following effects were also significant: *Difficulty*, *F*(1,77) = 94.07, *p* = < 0.001, η^2^ = 0.01, and Visualization * Item, *F*(1,77) = 8.22, *p* = 0.005, η^2^ = 0.003. Not significant were the main effect for *Item*, *F*(1,77) = 1.14, *p* = 0.29, η^2^ = 0.001, and the two interaction effects for *Item* * *Difficulty*, *F*(1,77) = 3.23, *p* = 0.076, η^2^ = 0.00 and Visualization * Item * Difficulty, *F*(1,77) = 0.97, *p* = 0.33, η^2^ = 0.00. Mean fixations and their standard deviations are detailed in Table 3. Again, we ran the same analysis for dwell times and found the same qualitative pattern of results (for details, see Supplement S2).

**Figure 6:**
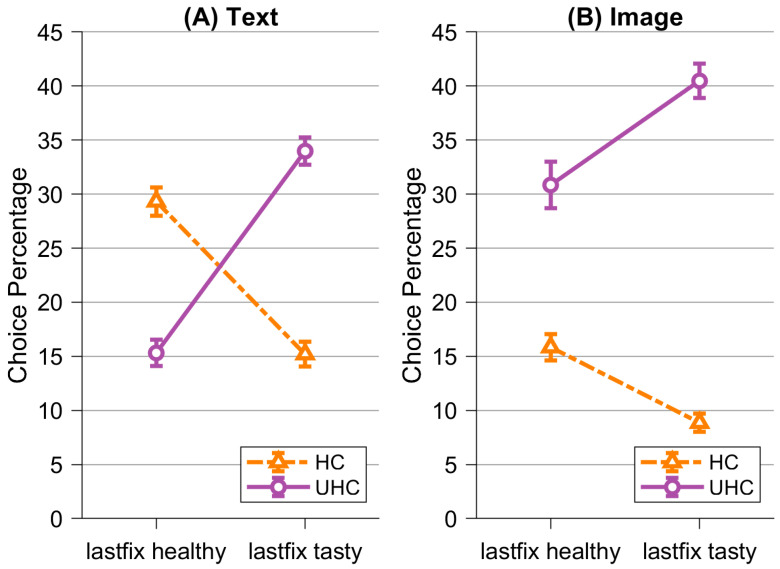
Choice percentages followed by last fixation of healthy item (lastfix healthy) and last fixation of tasty item (lastfix tasty) divided into healthy choices (HC, i.e., the healthy item was chosen) and unhealthy choices (UHC, i.e., the tasty item was chosen). (**A**) shows results for the text condition, while (**B**) shows results for the image condition. Error bars represent standard errors.

As a second exploratory analysis, we tested whether the last fixation influenced the subsequent choice (see Figure 6). For this purpose, we calculated rmANOVA including the factors *Visualization* (text; image), *Choice* (healthy; unhealthy), and *Last Fixation* (healthy item; tasty item). The dependent variable was the percentage of choices in relation to all choices in the visualization condition. We found a significant main effect for *Choice*, *F*(1,77) = 85.09, *p* < 0.001, η^2^ = 0.00. The other two main effects were not significant (*Visualization*: *F*(1,77) = 3.60, *p* = 0.062, η^2^ = 0.16; *Last Fixation*: *F*(1,77) = 2.55, *p* = 0.11, η^2^ = 0.00). Furthermore, we found a significant interaction effect for Visualization * Choice, F(1,77) = 136.61, *p* < 0.001, η^2^ = 0.11; Choice * Last Fixation, F(1,77) = 77.61, *p* < 0.001, η^2^ = 0.15; and Visualization * Choice * Last Fixation, *F*(1,77) = 21.60, *p* < 0.001, η^2^ = 0.02. In both conditions, the last fixation influenced the eventual choice such that participants made more healthy choices when the last fixation was on the healthy item as compared to when the last fixation was on the tasty item (and vice versa for unhealthy tasty choices). However, this effect was smaller in the image condition, where participants had a lower proportion of healthy compared to unhealthy choices even when the last fixation was on the healthy item. In contrast, in the text condition, participants’ choice percentages reversed according to their last fixations, such that participants made more healthy than unhealthy choices when the last fixation was on the healthy item and more unhealthy than unhealthy choices when the last fixation was on the tasty item. Mean choice percentages and their standard deviations are detailed in Table 4.

## 4. Discussion

In the present study, we examined how the framing of food options affects decision conflict and visual attention in food choices. Participants made binary choices between food items that were healthy but less tasty or less healthy but tasty (based on participants’ individual ratings). The food items were presented either as a typical food choice task in the form of images (e.g., an apple; image condition) or pre-matched value-based percentages of health and taste properties (e.g., 90% healthy and 20% tasty; text condition). At the behavioral level, we found a lower proportion of healthy choices and lower response times in the image condition compared to the text condition, replicating our previous findings [22]. In terms of visual attention, the results showed more fixations and longer dwell times on the item corresponding to the subsequent choice in the text condition (i.e., on the healthy item for healthy choices and on the tasty item for unhealthy choices). In the image condition, however, this only applies to the healthy item.

The current findings indicate that the presentation of food items as images, as is typical for food choice tasks, induces a greater temptation to opt for the unhealthy choice compared to a mere trade-off choice based on health and taste values. Participants showed a significantly higher preference for the tasty (i.e., unhealthy) option in the image condition than in the text condition. Furthermore, we found lower response times in the image condition compared to the text condition. There are two possible explanations for this finding: Either a generally faster decision-making process leads to more impulsive decisions, or there are general differences in the decision making process between the conditions.

The assumption of a faster decision-making process is supported by the fact that pictorial information is processed faster [18,19], potentially resulting in faster decision-making and consequently more impulsive selections [42]. Moreover, in the text condition, where both health and taste information were explicitly presented, the need for explicit processing of additional information could lead to extended decision times. The prolonged processing in itself causes longer decision times and could contribute to less impulsive choices. Furthermore, previous research has demonstrated that taste is processed more rapidly and therefore has a greater influence on decisions [11,43], which could support more impulsive decisions.

However, there are also some indications suggesting that it is not only a matter of faster and hence more impulsive decisions in the image condition, but that the decision processes differ between both conditions. First, in the text condition, we found effects in the response times that are comparable with prior research concerning value-based and rational choice results, like longer response times for difficult choices [44]. This is to be expected for a trade-off between attributes of two options. Interestingly, this effect was much smaller in the image condition. In addition, the attention processes seem to differ between the two conditions. In the text condition, results show more fixations in difficult trials [45] and longer dwell times for difficult decisions. Furthermore, we found evidence for the gaze cascade effect in the text condition, which refers to the tendency for the course of decision-making attention to shift to the chosen option [46,47]. For example, we found more fixations and longer dwell times on the item corresponding to the subsequent choice, and the last fixation of an item led to a corresponding choice on average (i.e., the healthy choice if the healthy item was fixated last and the unhealthy choice if the tasty item was fixated last). In the image condition, however, we did not find differences in response times as a function of difficulty and no differences in fixations and dwell time between difficult and easy decisions. Moreover, only minor evidence of the gaze cascade effect [47], according to which last fixation predicts choice, was found in this condition. Taken together, this suggests that typical food choice tasks that present food items as images go beyond a trade-off of health and taste attributes. Thereby, participants deviate from their own subjective trade-off between health and taste, as assessed in the task condition. Instead, when food options are presented as images, the tasty option seems to influence participants’ attention and decision-making processes quite strongly, as evidenced by the tasty item receiving the same attention in healthy decisions as in unhealthy decisions, and participants choosing the tasty option even if their last fixation was on the healthy option.

As different decision strategies might be involved in both conditions, an interesting question for future studies is whether an option-based or attribute-based strategy is used for decision-making in the text condition. These two approaches to decision-making are discussed in the existing body of research on classical intertemporal choice tasks [48,49,50,51]. In the option-based approach, it is posited that a subjective value is derived for each option by considering both the amount and time associated with it, and the option with the higher subjective value is subsequently chosen. Conversely, the attribute-wise approach contends that decisions are made through a direct comparison of attributes, such as value versus value and time versus time. When applied to the context of food choice in our study, both decision strategies, namely the option-based approach (where subjective values are compared) and the attribute-based approach (where attributes are directly compared), can be employed in the text condition. However, in the image condition, the health and taste attributes are already presented in an integrated manner, suggesting a predisposition towards an option-based decision-making process. This aspect may have also contributed to the observed differences between the two conditions. However, this could not be investigated in the present study, as we presented the health and taste values close to each other to achieve comparable stimulus sizes between the text and image condition.

Our findings have important implications for the field of food choice research. Our results suggest that decision-making in food choices does not merely reflect a trade-off between the health and taste properties of each food item. This could explain why people sometimes make dietary choices that are inconsistent with their own dietary goals and beliefs (e.g., preferring a tasty but less healthy option even though healthiness of food items matters to them). Our results also offer suggestions on how to promote healthy food choices. First, when participants have to make an abstract trade-off between health and taste attributes of two food items, they make a higher number of healthy choices than when matching food items are presented as images. This suggests that the representation of food as an image should be avoided in order to promote healthy choices, e.g., in school cafeterias or canteens. However, prior literature also shows that simultaneous presentation of image and additional information (e.g., caloric information, tastiness of healthy items) leads to more healthy choices [9,16]. Future studies could investigate the question as to whether it is solely the speed of decision-making that leads to more impulsive decisions with visual stimuli, or if it is specifically the stronger weighting of taste-related information. This could be investigated, for instance, by either imposing a minimum decision-making time or by comparing the effects of potentially relevant information (such as caloric information) with potentially irrelevant information (such as unrelated text) when presented concurrently with the visual stimulus. Second, the results suggest that visual attention has a different role in the image condition than in the text condition, but still has an effect on the decision process. This could indicate that healthy choices can be promoted by controlling visual attention (e.g., through salience of the stimuli or through presentation order). This is supported by a study by Dai et al. [52], who showed that perceptual salience can influence food choices irrespective of health and taste preferences. Therefore, making healthy food items more salient when presenting them visually might increase healthy food choices.

Some limitations should be considered when interpreting the results of the current study. First, the stimulus material for the two task conditions was very different. While the typical food choice task consisted of images, the value-based trade-off task consisted of abstract numerical health and taste attributes. It could be argued that this abstract presentation is too far removed from realistic food choices to draw conclusions about decision making in food choices. However, as such a trade-off is often assumed to be the basis for food choices, it is important to explicitly investigate this question. Future studies could build on this line of research by comparing the value-based trade-off task with a text-based food choice task (i.e., where food stimuli are presented as words instead of images).

Second, as outlined above, we presented health and taste values together as one area of interest. While this was necessary to achieve a comparable setup to the image condition, this limits the research questions that can be addressed in our experiment. In future studies, both properties could be presented in such a way that the fixations and gaze duration for the individual properties can be examined separately. This would allow us to gain further insights in to potential differences in option-based vs. attribute-based decision-making strategies.

## 5. Conclusions

The current study sheds light on how presenting food options as images (as is the norm for food choice tasks; image condition) or as abstract percentages of health and taste values (text condition) affects the underlying decision-making processes. Our findings reveal that presenting food options as images results in fewer healthy food choices, accompanied by lower response times and therefore more impulsive decision-making. Additionally, attentional processes differ between the two conditions: in the text condition, the item corresponding to the subsequent choice receives more attention than the alternative option. On the contrary, in the image condition, this only applies to the healthy item, indicating that participants divert from their own subjective health-taste trade-off when food options are presented as images. This implicates that food choices go beyond a mere value-based trade-off due to an increased attentional bias towards the tasty option. Rather, presenting food choices as images appears to trigger a different decision-making process that leads to fewer healthy choices, highlighting that the presentation format of food options is an important factor in promoting healthier food choices.

## Figures and Tables

**Table 1:** Descriptive statistics for response times.

Condition	Mean RT (s)	*SD* (s)
Easy healthy (text)	1.51	0.36
Easy unhealthy (text)	1.49	0.36
Difficult healthy (text)	1.72	0.44
Difficult unhealthy (text)	1.70	0.44
Easy healthy (image)	1.07	0.29
Easy unhealthy (image)	0.93	0.24
Difficult healthy (image)	1.15	0.28
Difficult unhealthy (image)	0.96	0.23

Note: RT = response time, *SD* = standard deviation.

**Table 2:** Mean fixations (fix) and their standard deviations (*SD*) divided into healthy choices (HC) vs. unhealthy choices (UHC), healthy item (HI) vs. tasty item (TI), and text vs. image condition.

Condition	Mean *n* Fixations	*SD*
HC HI (text)	3.27	0.88
HC TI (text)	2.48	0.88
UHC HI (text)	2.62	0.81
UHC TI (text)	3.06	0.97
HC HI (image)	1.89	0.53
HC TI (image)	1.57	0.45
UHC HI (image)	1.39	0.36
UHC TI (image)	1.60	0.47

**Table 3:** Mean fixations (fix) and their standard deviations (*SD*) on healthy (HI) and tasty items (TI) for both difficulties (easy vs. difficult) and both conditions (text vs. image).

Condition	Mean Fix	*SD*
Easy HI (text)	2.71	0.74
Easy TI (text)	2.51	0.77
Difficult HI (text)	3.01	0.92
Difficult TI (text)	2.88	0.99
Easy HI (image)	1.48	0.40
Easy TI (image)	1.53	0.43
Difficult HI (image)	1.51	0.41
Difficult TI (image)	1.57	0.41

**Table 4:** Mean choice percentages and their standard deviations (*SD*) for last fixation on healthy item (lastfix HI) and last fixation on tasty item (lastfix TI), divided into conditions (text vs. image), and in healthy choices (HC) and unhealthy choices (UHC).

Condition	Mean Choice (%)	*SD* (%)
HC lastfix HI (text)	29.33	11.55
HC lastfix TI (text)	15.20	10.16
UHC lastfix HI (text)	15.31	10.68
UHC lastfix TI (text)	33.98	11.15
HC lastfix HI (image)	15.85	10.76
HC lastfix TI (image)	8.86	7.34
UHC lastfix HI (image)	30.84	19.07
UHC lastfix TI (image)	40.47	13.97

## Data Availability

The datasets and analysis scripts supporting the conclusions of this article are openly available in the OSF repository, https://osf.io/7qmdc/.

## Supplementary Materials

The following supporting information can be downloaded at: https://www.mdpi.com/article/10.3390/nu16193343/s1. Supplement S1: Mean dwell times on healthy Item (healthy) and tasty Item (tasty), divided into healthy choices (HC) and unhealthy choices (UHC). (A) shows results for the text condition, while (B) shows results for the image condition. Supplement S2: Mean dwell times for both conditions (text vs. image), divided into healthy Item (healthy) and tasty Item (tasty), and easy decisions (easy) and difficult decisions (difficult). Error bars represent standard errors.

## References

[1] Rangel A., Camerer C., Montague P.R. (2008). A Framework for Studying the Neurobiology of Value-Based Decision Making. Nat. Rev. Neurosci..

[2] Jocham G., Hunt L.T., Near J., Behrens T.E.J. (2012). A Mechanism for Value-Guided Choice Based on the Excitation-Inhibition Balance in Prefrontal Cortex. Nat. Neurosci..

[3] Lin H., Saunders B., Hutcherson C.A., Inzlicht M. (2018). Midfrontal Theta and Pupil Dilation Parametrically Track Subjective Conflict (but Also Surprise) during Intertemporal Choice. Neuroimage.

[4] Shenhav A., Buckner R.L. (2014). Neural Correlates of Dueling Affective Reactions to Win-Win Choices. Proc. Natl. Acad. Sci. USA.

[5] Guh D.P., Zhang W., Bansback N., Amarsi Z., Birmingham C.L., Anis A.H. (2009). The Incidence of Co-Morbidities Related to Obesity and Overweight: A Systematic Review and Meta-Analysis. BMC Public Health.

[6] Tremmel M., Gerdtham U.G., Nilsson P.M., Saha S. (2017). Economic Burden of Obesity: A Systematic Literature Review. Int. J. Environ. Res. Public Health.

[7] Stroebe W., Van Koningsbruggen G.M., Papies E.K., Aarts H. (2013). Why Most Dieters Fail but Some Succeed: A Goal Conflict Model of Eating Behavior. Psychol. Rev..

[8] Diao L., Li W., Zhang W., Ma Q., Jin J. (2021). Electroencephalographic Theta-Band Oscillatory Dynamics Represent Attentional Bias to Subjective Preferences in Value-Based Decisions. Psychol. Res. Behav. Manag..

[9] Lim S.L., Penrod M.T., Ha O.R., Bruce J.M., Bruce A.S. (2018). Calorie Labeling Promotes Dietary Self-Control by Shifting the Temporal Dynamics of Health- and Taste-Attribute Integration in Overweight Individuals. Psychol. Sci..

[10] Stillman P.E., Medvedev D., Ferguson M.J. (2017). Resisting Temptation: Tracking How Self-Control Conflicts Are Successfully Resolved in Real Time. Psychol. Sci..

[11] Sullivan N.J., Hutcherson C., Harris A., Rangel A. (2015). Dietary Self-Control Is Related to the Speed with Which Attributes of Healthfulness and Tastiness Are Processed. Psychol. Sci..

[12] Hare T.A., Camerer C.F., Rangel A. (2009). Self-Control in Decision-Making Involves Modulation of the VmPFC Valuation System. Science.

[13] Kruse J., Korb F.M., Surrey C., Wolfensteller U., Goschke T., Scherbaum S. (2024). Focusing on Future Consequences Enhances Self-Controlled Dietary Choices. Nutrients.

[14] Undarwati A., Why F.Y.P. (2024). BMI and Explicit-Implicit Cues on Food Choice: The Fake Food Buffet in the United Kingdom and Indonesia. Appetite.

[15] Gillebaart M., Blom S.S.A.H., Benjamins J.S., de Boer F., De Ridder D.T.D. (2023). The Role of Attention and Health Goals in Nudging Healthy Food Choice. Front. Psychol..

[16] Li X., Ruan Y., Wang S., Pan Y., Huang Y. (2024). Taste or Health: The Impact of Packaging Cues on Consumer Decision-Making in Healthy Foods. Appetite.

[17] Yang X., Krajbich I. (2023). A Dynamic Computational Model of Gaze and Choice in Multi-Attribute Decisions. Psychol. Rev..

[18] Hogaboam T.W., Pellegrino J.W. (1978). Hunting for Individual Differences in Cognitive Processes: Verbal Ability and Semantic Processing of Pictures and Words. Mem. Cognit..

[19] Potter M.C., Faulconer B.A. (1975). Time to Understand Pictures and Words. Nature.

[20] Burnett Heyes S., Adam R.J., Urner M., van der Leer L., Bahrami B., Bays P.M., Husain M. (2012). Impulsivity and Rapid Decision-Making for Reward. Front. Psychol..

[21] Dshemuchadse M., Scherbaum S., Goschke T. (2013). How Decisions Emerge: Action Dynamics in Intertemporal Decision Making. J. Exp. Psychol. Gen..

[22] Kruse J., Senftleben U., Scherbaum S., Korb F.M. (2024). A Picture Is Worth a Thousand Words: Framing of Food Choice Options Affects Decision Conflict and Mid-Fontal Theta in Food Choice Task. Appetite.

[23] Schotter E.R., Berry R.W., McKenzie C.R.M., Rayner K. (2010). Gaze Bias: Selective Encoding and Liking Effects. Vis. Cogn..

[24] Danner L., De Antoni N., Gere A., Sipos L., Kovács S., Dürrschmid K. (2016). Make a Choice! Visual Attention and Choice Behaviour in Multialternative Food Choice Situations. Acta Aliment..

[25] Krajbich I., Armel C., Rangel A. (2010). Visual Fixations and the Computation and Comparison of Value in Simple Choice. Nat. Neurosci..

[26] van der Laan L.N., Hooge I.T.C., de Ridder D.T.D., Viergever M.A., Smeets P.A.M. (2015). Do You like What You See? The Role of First Fixation and Total Fixation Duration in Consumer Choice. Food Qual. Prefer..

[27] Corbetta M., Shulman G.L. (2002). Control of Goal-Directed and Stimulus-Driven Attention in the Brain. Nat. Rev. Neurosci..

[28] Rayner K., Miller B., Rotello C.M. (2008). Eye Movements When Looking at Print Advertisements: The Goal of the Viewer Matters. Appl. Cogn. Psychol..

[29] Lohse G.L. (1997). Consumer Eye Movement Patterns on Yellow Pages Advertising. J. Advert..

[30] van der Laan L.N., Papies E.K., Hooge I.T.C., Smeets P.A.M. (2017). Goal-Directed Visual Attention Drives Health Goal Priming: An Eye-Tracking Experiment. Health Psychol..

[31] Milosavljevic M., Navalpakkam V., Koch C., Rangel A. (2012). Relative Visual Saliency Differences Induce Sizable Bias in Consumer Choice. J. Consum. Psychol..

[32] Ahn S.E., Oh J., Cho M.S. (2023). Capturing Consumers’ Visual Attention toward Sugar-Reduction Information—Focusing on Sugar-Reduced Beverages Using Eye-Tracking Experiments. Br. Food J..

[33] Gidlöf K., Anikin A., Lingonblad M., Wallin A. (2017). Looking Is Buying. How Visual Attention and Choice Are Affected by Consumer Preferences and Properties of the Supermarket Shelf. Appetite.

[34] Freijy T., Mullan B., Sharpe L. (2014). Food-Related Attentional Bias. Word versus Pictorial Stimuli and the Importance of Stimuli Calorific Value in the Dot Probe Task. Appetite.

[35] Czyzewska M., Graham R. (2008). Implicit and Explicit Attitudes to High- and Low-Calorie Food in Females with Different BMI Status. Eat. Behav..

[36] Oakes M.E., Slotterback C.S. (2002). The Good, the Bad, and the Ugly: Characteristics Used by Young, Middle-Aged, and Older Men and Women, Dieters and Non-Dieters to Judge Healthfulness of Foods. Appetite.

[37] Campbell J.I.D., Thompson V.A. (2012). MorePower 6.0 for ANOVA with Relational Confidence Intervals and Bayesian Analysis. Behav. Res. Methods.

[38] Greiner B. (2015). Subject Pool Recruitment Procedures: Organizing Experiments with ORSEE. J. Econ. Sci. Assoc..

[39] Brainard D.H. (1997). The Psychophysics Toolbox. Spat. Vis..

[40] Blechert J., Meule A., Busch N.A., Ohla K. (2014). Food-Pics: An Image Database for Experimental Research on Eating and Appetite. Front. Psychol..

[41] JASP Team (2019). JASP (Version 0.10.2.0); [Computer Software]. https://jasp-stats.org/.

[42] Edman G., Schalling D., Levander S.E. (1983). Impulsivity and Speed and Errors in a Reaction Time Task: A Contribution to the Construct Validity of the Concept of Impulsivity. Acta Psychol..

[43] Köster M., Buabang E.K., Ivančir T., Moors A. (2023). A Value Accumulation Account of Unhealthy Food Choices: Testing the Influence of Outcome Salience under Varying Time Constraints. Cogn. Res. Princ. Implic..

[44] Atzori R., Pellegrini A., Lombardi G.V., Scarpa R. (2023). Response Times and Subjective Complexity of Food Choices: A Web-Based Experiment Across 3 Countries. Soc. Sci. Comput. Rev..

[45] Young A.H., Hulleman J. (2013). Eye Movements Reveal How Task Difficulty Moulds Visual Search. J. Exp. Psychol. Hum. Percept. Perform..

[46] Fiedler S., Glöckner A. (2012). The Dynamics of Decision Making in Risky Choice: An Eye-Tracking Analysis. Front. Psychol..

[47] Shimojo S., Simion C., Shimojo E., Scheier C. (2003). Gaze Bias Both Reflects and Influences Preference. Nat. Neurosci..

[48] Amasino D.R., Sullivan N.J., Kranton R.E., Huettel S.A. (2019). Amount and Time Exert Independent Influences on Intertemporal Choice. Nat. Hum. Behav..

[49] Khaw M.W., Li Z., Woodford M. (2018). Temporal Discounting and Search Habits: Evidence for a Task-Dependent Relationship. Front. Psychol..

[50] Liu H.Z., Lyu X.K., Wei Z.H., Mo W.L., Luo J.R., Su X.Y. (2021). Exploiting the Dynamics of Eye Gaze to Bias Intertemporal Choice. J. Behav. Decis. Mak..

[51] Reeck C., Wall D., Johnson E.J. (2017). Search Predicts and Changes Patience in Intertemporal Choice. Proc. Natl. Acad. Sci. USA.

[52] Dai J., Cone J., Moher J. (2020). Perceptual Salience Influences Food Choices Independently of Health and Taste Preferences. Cogn. Res. Princ. Implic..

