# Supplementary material for: You Eat with Your Eyes: Framing of Food Choice Options Affects Decision Conflict and Visual Attention in Food Choice Task
Source: Nutrients. 2024 Oct 1;16(19):3343. doi: 10.3390/nu16193343 (PMC11478952; doi:10.3390/nu16193343)
Supplement: Supplementary file 1 [file nutrients-16-03343-s001.zip › nutrients-3197879-supplementary.pdf]

Supplement for "You eat with your eyes: Framing of food choice options affects decision conflict and visual attention in food choice task"

Ulrike Senftleben, Johanna Kruse, Stefan Scherbaum & Franziska M. Korb

Corresponding author: Ulrike Senftleben

ulrike.senftleben@tu-dresden.de

ORCID: 0000-0002-8403-0359

Technische Universität Dresden

Professur Methoden der Psychologie und kognitive Modellierung

01062 Dresden

Germany

S1)

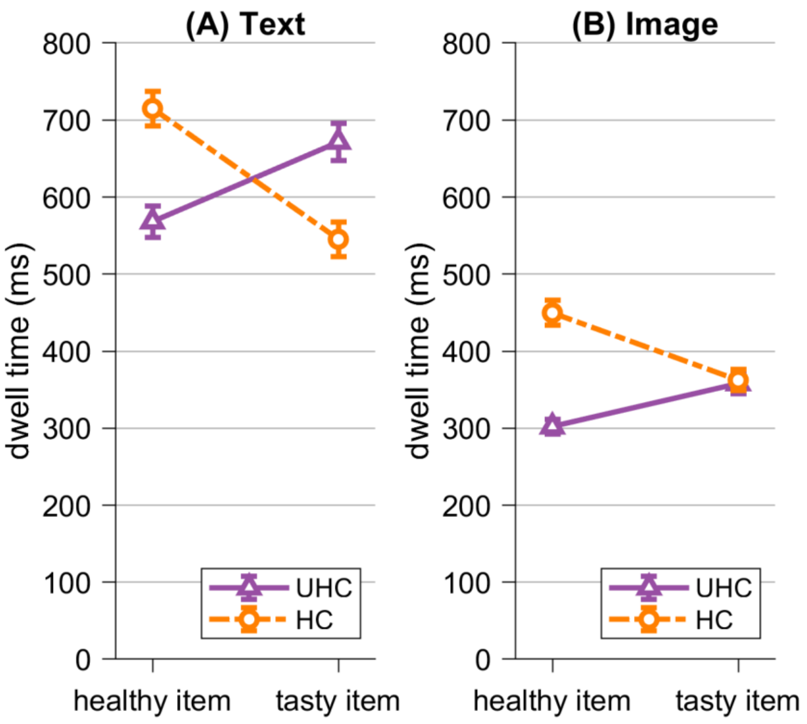

Figure S1. Mean dwell times on healthy Item (healthy) and tasty Item (tasty), divided into healthy choices (HC) and unhealthy choices (UHC). (A) shows results for the text condition, while (B) shows results for the image condition. Error bars represent standard errors.

We found a significant main effect for *Visualization*, $F(1,77) = 246.24$, $p < .001$, $\eta^2 = .35$. Furthermore, we found a significant main effect for *Choice* ($F(1,77) = 35.93$, $p = < .001$, $\eta^2 = .01$), and *Item* ($F(1,77) = 5.21$, $p = .025$, $\eta^2 = .003$). All interaction effects were significant (*Item* * *Choice*, $F(1,77) = 239.60$, $p < .001$, $\eta^2 = .06$; *Visualization* * *Choice*, $F(1,77) = 34.76$, $p < .001$, $\eta^2 = .006$; *Visualization* * *Item* * *Choice*, $F(1,77) = 78.76$, $p < .001$, $\eta^2 = .006$), except for *Visualization* * *Item*, $F(1,77) = 1.14$, $p = .29$, $\eta^2 = .00$.

S 2)

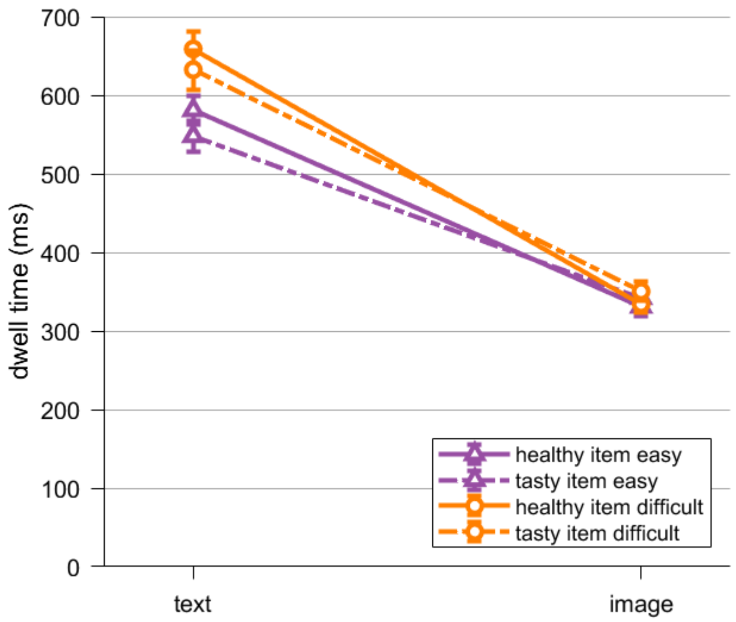

Figure S2. Mean dwell times for both conditions (text vs. image), divided into healthy Item (healthy) and tasty Item (tasty), and easy decisions (easy) and difficult decisions (difficult). Error bars represent standard errors.

We found a significant main effect for *Visualization*, $F(1,77) = 274.18$, $p < .001$, $\eta^2 = .42$. The dwell time was higher for items in the text condition (605.13 ms, $SD = 196.30$ ms) as compared to the image condition (339.35 ms, $SD = 103.95$ ms). Furthermore, we found a significant main effect for *Difficulty* ($F(1,77) = 99.487$, $p = < .001$, $\eta^2 = .01$). Dwell time was higher for items when the decision was difficult (494.02 ms, $SD = 224.48$ ms) as compared to the easy decisions (450.58 ms, $SD = 182.76$ ms). The main effect *Item* was not significant ($F(1,77) = .60$, $p = .44$, $\eta^2 = .000$), but we found a significant interaction effect *Visualization* * *Item*, $F(1,77) = 7.15$, $p = .009$, $\eta^2 = .003$. As hypothesized, while dwell time was higher for the healthier item in the text condition, it was higher for the tastier item in the image condition. We also found a significant interaction effect *Visualization* * *Difficulty*, $F(1,77) = 66.37$, $p < .001$, $\eta^2 = .008$. Descriptively, dwell time was higher for the difficult item compared to the easy item in the text condition, whereas there is no difference in the image condition. The other two interaction effects were not significant (*Item* * *Difficulty*, $F(1,77) = 1.25$, $p = .27$, $\eta^2 = .00$; *Visualization* * *Item* * *Difficulty*, $F(1,77) = .01$, $p = .91$, $\eta^2 = .00$).
